# *Bacillus* spp. Spores—A Promising Treatment Option for Patients with Irritable Bowel Syndrome

**DOI:** 10.3390/nu11091968

**Published:** 2019-08-21

**Authors:** Adrian Catinean, Adriana Maria Neag, Andreea Nita, Mihaela Buzea, Anca Dana Buzoianu

**Affiliations:** 1 Department of Internal Medicine, Iuliu Hatieganu University of Medicine and Pharmacy, 400006 Cluj-Napoca, Romania; 2 Pharmacology, Toxicology and Clinical Pharmacology Department, Iuliu Hatieganu University of Medicine and Pharmacy, 400337 Cluj-Napoca, Romania; 3 Department of Gastroenterology, Octavian Fodor Regional Institute of Gastroenterology and Hepatology, 400162 Cluj-Napoca, Romania; 4 Diasan Medical Center, 400516 Cluj-Napoca, Romania

**Keywords:** irritable bowel syndrome, FODMAP, *Bacillus* spores, probiotics, rifaximin

## Abstract

Dysbiosis is a condition that can cause various clinical disorders, from gastrointestinal problems to allergies or even cancer. Resetting the microbiota using antibiotics and/or probiotics could be a possible therapy for many diseases. The aim of this study was to evaluate the effects of three treatment regimens in patients with irritable bowel syndrome (IBS). The regimens were short-term rifaximin treatment (10 days) followed by either a nutraceutical agent (G1) or a low- Fermentable, Oligo-, Di-, Monosaccharide and Polyol (FODMAP) diet (24 days) (G3) or treatment with MegaSporeBiotic a mixture of spores of five *Bacillus* spp. for medium-term (34 days) (G2). Ninety patients with IBS without constipation were enrolled and divided into three groups (G1, G2, G3). Patients in G1 and G3 were evaluated over four visits (baseline/first day (V1), 10 days (V2), 34 days (V3), 60 days (V4)), and, those in G2 over three visits (V1, V3, V4). Severity score, quality of life, and parameters from the rectal volume sensation test were determined. The results demonstrated that patients treated with MegaSporeBiotic, compared with those treated with rifaximin followed by nutraceutical or low-FODMAP diet, had similar severity scores and rectal volume sensation test results for all parameters tested and statistically significant improvement in measurements of quality of life.

## 1. Introduction

Irritable bowel syndrome (IBS) is an intestinal functional disorder characterized by recurrent abdominal pain or discomfort in association with changes in consistency or frequency of stool (diarrhea or constipation). This definition is in line with the Rome III criteria, because the new criteria (Rome IV) have excluded this parameter. The natural history of the disease includes both periods of relapse and remission [1,2].

Although many studies have investigated IBS, the pathophysiology remains unclear and controversial. Several mechanisms have been extensively investigated ranging from inflammation and immunological involvement to dysbiosis, brain–gut interaction, genetic or dietary factors [3,4,5,6,7]. Therapeutic management of IBS focuses on treatment to relieve symptoms, which is frequently unsatisfactory. Therefore, in addition to standard pharmacological treatment, alternative/integrative therapeutic approaches are needed.

One hypothesis is that overgrowth of certain bacteria in the gut can cause fermentation resulting in overproduction of gas, alteration of gastrointestinal motility and disruption of the mucosal barrier [8,9]. Considering this hypothesis, it would be possible to alleviate IBS symptoms in three ways: (1) Reducing the number of pathogenic bacteria using antibiotics [10,11]; (2) restoring intestinal homeostasis with probiotics [12]; and (3) consuming a low- Fermentable, Oligo-, Di-, Monosaccharide and Polyol (FODMAP) diet which contributes to the relief of gastrointestinal symptoms by reducing fermentable nutrients in the colon, gas production and abdominal distension. However, sometimes this type of diet can be dangerous, because it can cause nutrient deficiency or reduce the amount of fiber that are the substrate for microbiome development [13,14].

However, there are some limitations of these treatment methods. Due to the risk of both side effects and development of resistance, antibiotics have not been extensively used for IBS. Moreover, antibiotic use is associated with a reduction in gut microbial species such as archaea and metanobacter, generally responsable for small intestinal bacterial overgrowth (SIBO). Nevertheless, rifaximin is an antibiotic with a grade 2 recommendation and level of evidence grade B in patients with diarrhea-IBS, likely because it acts primarily in the gut and has low bioavailability and poor absorption [15]. Probiotics have a beneficial effect on the host by suppressing pathogens, producing bacteriocins and neurotransmitters and improving intestinal barrier function [16].

IBS treatments include medication (antibiotics, antidiarrheal, laxatives, antispastics), nutraceuticals (probiotics, prebiotics, symbiotics), diet, lifestyle change or a combination of these methods. There are limited studies comparing the efficacy of different treatment methods in patients with IBS. Additionally, the efficacy of conventional antibiotic combined with diet or nutritional agents versus probiotic spores has not yet been investigated in these patients.

The aim of the current study was to compare the effects of short-term treatment (10 days) with rifaximin, followed by 24 days of low-FODMAP diet or treatment with a nutraceutical agent containing a combination of prebiotics, probiotics and vitamins, with the effects of long-term treatment (34 days) with MegaSporeBiotic (100% spore-based probiotic containing *Bacillus licheniformis*, *Bacillus indicus HU36* ™, *Bacillus subtilis HU58* ™, *Bacillus clausii, Bacillus coagulans).*

## 2. Materials and Methods

### 2.1. Study Design and Patients

This was a prospective, randomized controlled clinical study conducted in a single medical center in Romania. The study protocol was approved by the institutional review board of Iuliu Hațieganu University of Medicine and Pharmacy, Cluj-Napoca, Romania (No. 18/2014), prior to patient recruitment. The study was conducted according to the principles of the Declaration of Helsinki of 1975, revised in 2013, and Good Clinical Practice guidelines.

### 2.2. Patients

Patients who presented with IBS at the Medical Center between March 2016 and March 2019 that met the inclusion criteria, did not meet the exclusion criteria and signed the informed consent were included in the study. The inclusion criteria were individuals diagnosed with non-constipation IBS based on Rome III criteria, no constipation, aged 18 to 75 years, normal colonoscopy in the last 5 years, blood counts within reference values and normal fecal calprotectin. We applied the Rome III criteria because we started the study before the Rome IV criteria were published (May 2016).

Patients with documented food allergies, gluten intolerance or celiac disease, diabetes, thyroid disease, intestinal inflammatory disease or other organic diseases, eating disorders (anorexia or bulimia), probiotics one month before the study, antibiotic treatment in the previous 6 months or those using specific diets (e.g., vegetarian) were excluded. Patients were not excluded if they had used diarrheal treatment, laxatives and/or antispasmodic medications; however, patients were required to discontinue such medications at study enrollment and signed the informed consent.

### 2.3. Intervention

**Figure 1:**
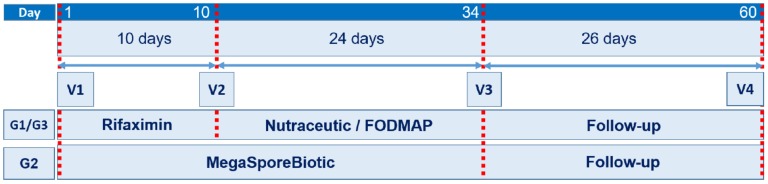
Study design. Treatment and follow-up periods with medical visits. Abbreviations: G = group, V = visit.

Enrolled patients were randomly assigned to one of three study groups (G1, G2 and G3). G1 and G3 were treated for 10 days with rifaximin (1200 mg daily) followed by treatment with a nutraceutical agent (probiotic—*Bifidobacterium longum W11* in combination with prebiotics—soluble fiber and group B vitamins—B1, B2, B6, B12) (G1) or a low-FODMAP diet (G3). G2 received a spore-based probiotic formulation consisting of five *Bacillus* spp. (MegaSporeBiotic, Microbiome Labs, St. Augustine, FL, USA) for 34 days (one capsule daily for the first 7 days followed by two capsules daily for 27 days). To increase the rate of eradication and extend the period of remission, treatment with rifaximin and a combination of probiotics or diet are recommended in patients with IBS [17]. Patients in G1 and G3 were evaluated at baseline/first day of the study (V1), 10 days (V2), 34 days (V3) and 60 days (V4, end of study) after study initiation. Patients in G2 were evaluated at V1, V3 and V4 (Figure 1).

### 2.4. Randomization

Patients were randomized equally (1:1:1) into each of the three groups. Each patient included in the study received their assigned treatment according to the schedule for a period of 2 months, treatment was initiated at (V1). This was an open-label study.

### 2.5. Measurements and Study Endpoints

#### 2.5.1. IBS Severity Score (IBS-SS)

To determine the IBS severity score (IBS-SS), patients completed a questionnaire developed by Francis et al. [18], which examined severity of abdominal pain, number of days with abdominal pain, distension and the impact of each on quality of life (QL). Each question had a score range from 0 (not at all impaired) to 100 (extremely impaired) and the questionnaire could have a total score of 0 to 500, with 500 being the highest possible IBS-SS score. Higher scores are correlated with greater severity of symptoms. Mild, moderate and severe cases are indicated by scores of 75–175, 175–300 and more than 300, respectively [18].

#### 2.5.2. Quality of Life for IBS Patients (IBS-QL)

Quality of life (QL) was evaluated using the irritable bowel syndrome questionnaire which has 36 items (SF-36). It measures the patient’s state of health on eight points: Functional status (physical and social functioning, physical and emotional problems), well-being (mental health, vitality and pain) and general health assessment (general perception of health) [19,20].

Both questionnaires (IBS-SS and IBS-QL) were completed by patients in the presence of apsychologist.

#### 2.5.3. Rectal Volume Sensation Test

For the rectal volume sensation test we used a single use 8 channel anorectal catheter (4.9 mm 2300E, Mediplus, UK) with a universal thermoplastic balloon that expands from 60 to 400 mL attached to a Sandhill Insight manometry system. The patients received a sodium phosphate solution to clean the lower rectum before testing. Patients were placed in a left lateral position and the lubricated barista bag was slowly inserted into the rectum. Patients had 2–3 min to adapt, then the bag was progressively inflated with air. The thresholds for the first sensation of swollen balloon, tenesmus and discomfort were indicated by the patients. Every perceived sensation was correlated with sphincter relaxation. The balloon was inflated until the patient first reported pain [21,22]. The test was performed on all patients by the same medical team.

#### 2.5.4. Statistical Analysis

IBM SPSS version 22 (IBM Corp., Armonk, NY, USA) software was used for statistical analyses. Descriptive statistics were calculated for all variables. Data are shown as mean ± standard deviation (SD). Intra- and intergroup differences before/during/after therapy were analyzed. For longitudinal intragroup comparisons between consecutive measurements in the same sample of patients, multiple paired-samples t-tests for dependent variables were used. ANOVA and Bonferroni post-hoc tests for independent variables were applied when comparisons were made between groups. The threshold for statistical significance was considered as *p* > 0.05.

## 3. Results

### 3.1. Patients

In total, 90 patients were enrolled in the study, 30 in each group. Demographic characteristics are shown in Table 1. There were (n (%)) 16 (53.3%), 21 (70%) and 17 (56.7%) women in G1, G2 and G3, respectively. Mean ± SD for body mass index (BMI) was 25.32 ± 4.32 kg/m^2^ for G1, 24.73 ± 6.19 kg/m^2^ for G2, and 25.65 ± 4.76 kg/m^2^ for G3. There were no statistically significant differences in age or BMI among the groups.

### 3.2. IBS-SS

#### 3.2.1. Intragroup Results for IBS-SS

The IBS-SS results for each group are shown in Table 2.

Compared to the IBS-SS, a statistically significant difference between successive visits (V1 vs. V2, V2 vs. V3 and V3 vs. V4) was observed for all groups (G1, G2 and G3) (Table 3).

#### 3.2.2. Intergroup IBS-SS Results

There was an overall statistically significant difference between groups for the IBS-SS measurement at V3 (SS_V3; *p* = 0.028). We also compared pairs of groups (example: G1 vs. G2) using the Bonferroni post-hoc test to establish which ones differed for SS_V3. The results showed that there was a statistically significant difference between G2 and G1, but not between G2 and G3 (Table 4).

#### 3.2.3. IBS-QL Results

At each visit, we compared the scores from the IBS-QL questionnaire (SF-36) for general health (GH), physical functioning (PhF) and physical role functioning (PhRF).

There was an overall statistically significant mean difference between G1 and G3 vs. G2 with regard to V3_GH, V3_PhF, V4_GH, V4_PhF. Post-hoc Bonferroni pair testing was performed to identify the statistical significance of differences between pairs of groups. Both G1 vs. G2 and G2 vs. G3 had statistically significant differences with regard to the previously mentioned measurements (Table 5).

### 3.3. Rectal Volume Sensation Test

#### Intragroup Results

We evaluated whether there was a significant difference in the rectal volume test results between the four visits. As the group measurements represent the same patients followed throughout the treatment (paired), we applied a t-test for dependent variables (paired-samples *t*-test).

Measurements are noted as GxViy for x = 1 to 3 (corresponding to group number), i = 1 to 4 (corresponding to visit number) and y = 1 to 3 (corresponding to each parameter measured in the rectal sensation volume test which were: First sensation (FS), tenesmus (T) and pain (P), respectively).

By comparing the three parameters of the rectal volume sensation test measured at each visit (V1, V2, V3, V4) several statistically significant differences were found within G1: G1_V1_FS vs. G1_V2_FS (*p* < 0.05) G1_V1_T vs. G1_V2_T, G1_V2_T vs. G1_V3_T (*p* < 0.05, less highly significant than reported G1_V1_T vs. G1_V2_T) and G1_V1_P vs. G1_V2_P. No statistically significant difference was found for G1_V2_FS vs. G1_V3_FS (*p* > 0.05), G1_V3_T vs. G1_V4_T (*p* > 0.05) and G1_V2_P vs. G1_V3_P. For G2, we observed statistically significant differences between G2_V1_FS vs. G2_V3_FS, G2_V1_T vs. G2_V3_T and G2_V1_P vs. G2_V3_P but not between G2_V3_FS vs. G2_V4_FS, G2_V3_T vs. G2_V4_T and G2_V3_P vs. G2_V4_P. For G3, we observed statistically significant differences for G3_V1_FS vs. G3_V2_FS, G3_V1_T vs. G3_V2_T, G3_V1_P vs. G3_V2_P and G3_V2_P vs. G3_V3_P but not for G3_V2_FS vs. G3_V3_FS or G3_V2_T vs. G3_V3_T (Table 6).

There was a statistically significant difference between groups for the first measurement (FS) of the third (V3) rectal volume sensation test, but not for the second and third measurements (Table 7).

## 4. Discussion

In this study we compared the effects of treatment with a spore-based probiotic mixture of five *Bacillus* spp. (MegaSporeBiotic) on IBS-SS, IBS-QL and rectal volume sensation in IBS patients to two standard treatment regimens for these patients: Rifaximin followed by a nutraceutical agent (*Lactobacillus* strain, prebiotics and vitamins) or rifaximin followed by a low-FODMAP diet.

*Bacillus* spp. are of particular interest to humans due to their tolerance of and ability to survive in environments of gastric acidity or the hostile environment of the intestine. These microorganisms are also important for other animals as growth regulators and for providing protection against diseases in aquaculture [23]. Although *Bacillus* spp. have a high biotherapeutic potential for production of antimicrobial peptides, production of additional vitamins (e.g., cobalamin, riboflavin) and for modulating the host microbiota, studies of this species as probiotics have only been on the rise over the past 10 years [24]. Currently, *Bacillus* spp. are some of the most studied and well-characterized probiotics; their use as probiotics expanding rapidly because of their inherent ability to form endospores [25].

In our study, the mean IBS-SS ranged from 175 to 300 for all groups at V1. Thus, we can reasonably assume similar severity of disease in all groups (moderate disease) at the beginning of the study. IBS-SS decreased with each visit for all groups, all of the treatments tested had a positive impact on IBS-SS. Between groups (intergroup), significant difference were only observed at V3 (34 days). The results showed that treatment with *Bacillus* spores (G2) attenuated the IBS-SS score better than rifaximin plus the nutraceutical agent (G1) and similarly to rifaximin plus a low-FODMAP diet (G3). If the IBS is linked to dysbiosis, our results demonstrate that *Bacillus* spp. spore-based probiotics have the capacity to reduce gut dysbiosis to a similar degree as antibiotic treatment. The efficacy of rifaximin has been demonstrated in previous studies, but many of these were compared with placebo rather than a combination of treatments (rifaximin and other pharmacological or non-pharmacological agents) or with other therapies [10,26,27]. The positive effect of a low-FODMAP diet on the severity score in patients with IBS has been demonstrated in meta-analyses [28,29]. The efficacy of this treatment to alleviate IBS symptoms could be explained by its impact on SIBO. This condition may be a cause of IBS [30]. It is known that rifaximin is a clinically effective antibiotic in patients with SIBO, but rifaximin monotherapy may cause rapid relapse [17]. For this reason, we recommended rifaximin and either a nutraceutical agent (G1) or a low-FODMAP diet (G3). Rifaximin destroys certain bacteria, while the nutraceutical agent and low-FODMAP diet stimulate the growth of “good” bacteria restoring the intestinal microbial balance. This could be considered reset–recovery effect of gut microbiota.

*Bacillus* spp. are spore forming bacteria and thus more resistant to passage through the upper digestive tract than other probiotics [31]. Studies have shown that certain species of *Bacillus* spores are capable of quorum sensing. Through quorum sensing, they exert an important regulatory effect on intestinal microbiota resulting in positive effects on both the colon and ileum [32]. It is likely that the positive effect of MegaSporeBiotic is a consequence of both microbiota modulation and influence on the gut–brain axis through the intestinal enterochromaffin cells [33]. Regarding QL, we monitored general health, physical functioning and physical role functioning; we observed a statistically significant difference for these parameters in V3 and V4. The results showed that after 34 days of treatment, the impact of *Bacillus* spores on QL was superior to treatment with rifaximin combined with either a nutraceutical agent or a low-FODMAP diet. *Bacillus* spp. have the capacity to produce more extracellular molecules than *Lactobacillus*. This could explain the superior effect of MegaSporeBiotic observed in our study [34].

The parameters evaluated using the rectal volume sensation test showed that rifaximin had a beneficial effect on all the measured parameters (FS, T and P), while the nutraceutical agent only had a beneficial effect on T and the low-FODMAP diet had an effect on FS and P. However, at 34 days (V3), all of the treatments tested had beneficial effects on all parameters of the rectal volume sensation test.

The FS parameter of the rectal volume sensation test and those related to QL have a high degree of subjectivity. The results reported in this study may be due to modulation of the microbiota and the influence of the gut–brain axis.

## 5. Limitations

It is necessary to take into account certain limits of our study:

- The study was not a blind study, so both the patients and health professionals were aware of treatment. The parameters reported in our study are based on the patient’s report and therefore may been influenced by their knowledge of IBS and their assigned treatment.
- When the study was initiated, the patients were not severely symptomatic (mean IBS-SS < 300). Thus, questions regarding frequency of symptoms, rather than treatment severity, may have been more appropriate for the patient population and give more sensitive results.
- We applied Rome III criteria not Rome IV. Even though we applied the Rome III criteria, approximately 90% of the patients enrolled in the study met the Rome IV criteria for IBS.
- SIBO was not tested in the study patients.

## 6. Conclusions

Overall, our study demonstrated that MegaSporeBiotic (a mix of spores from five *Bacillus* spp.) had a similar impact on severity score and all parameters of the rectal volume sensation test and a significantly better effect on QL compared with rifaximin plus nutraceutical agent or a low-FODMAP diet. Manipulation of the gut microbiota is essential to treat IBS; this may be achieved through treatment with spore-based *Bacillus* spp. probiotics.

## Figures and Tables

**Table 1:** Patient demographics at baseline.

Parameters	G1 (Rifaximin/Nutraceutical)	G2 (MegaSporeBiotic)	G3 (Rifaximin/Low-FODMAP)
No. of participants	30	30	30
Sex, n (%)			
Male	14 (46.7%)	9 (30%)	13 (43.3%)
Female	16 (53.3%)	21 (70%)	17 (56.7%)
Age			
(mean ± SD)	38.77 ± 10.96	39.07 ± 16.00	40.37 ± 11.952
Weight (mean ± SD)	74.97 ± 15.92	70.97 ± 19.63	78.13 ± 19.22
Height (mean ± SD)	1.71 ± 0.075	1.69 ± 0.081	1.73 ± 0.085
BMI (mean ± SD)	25.32 ± 4.32	24.73 ± 6.19	25.65 ± 4.76

Abbreviations: G, group; SD, standard deviation; BMI, body mass index.

**Table 2:** IBS-SS for patients in all groups.

Visit	G1	G2	G3
	(Mean ± SD)		
V1	258.17 ± 69.07	268.33 ± 81.45	260.33 ± 83.11
V2	113.17 ± 41.55	-	120.83 ± 54.88
V3	89.33 ± 41.88	67.00 ± 17.25	85.67 ± 37.38
V4	40.00 ± 27.41	42.67 ± 24.30	47.83 ± 30.81

Abbreviations: G, group; V, visit; IBS-SS, inflammatory bowel syndrome severity score; SD, standard deviation.

**Table 3:** IBS-SS comparisons for each group.

Group	Pair	Paired Differences			*p*-Value
		Mean ± SD	95% Confidence Interval		
			Lower	Upper	
G1	SS_V1-SS_V2	145.00 ± 57.76	123.43	166.56	0.0001
	SS_V2-SS_V3	23.83 ± 44.52	7.20	40.45	0.007
	SS_V3-SS_V4	49.33 ± 29.96	38.14	60.52	0.0001
G2	SS_V1-SS_V3	201.33 ± 71.76	174.53	228.13	0.0001
	SS_V3-SS_V4	24.33 ± 18.08	17.58	31.08	0.0001
G3	SS_V1-SS_V2	139.50 ± 59.91	117.12	161.87	0.0001
	SS_V2-SS_V3	35.16 ± 42.17	19.42	50.91	0.0001
	SS_V3-SS_V4	37.83 ± 29.20	26.92	48.73	0.0001

A *p*-value < 0.05 was considered significant. Abbreviations: SS_Vn, severity score at visit n; G, group; SD, standard deviation.

**Table 4:** Comparison of severity score of G2 (MegaSporeBiotic) with G1 and G3.

Dependent Variable	(I) (1 = G1, 2 = G2, 3 = G3)	(II) (1 = G1, 2 = G2, 3 = G3)	Mean Difference (I–II)	*p*-Value
SS_V3	2	1	−22.333	0.038
		3	−18.667	0.108

A *p*-value < 0.05 was considered significant. Abbreviations: SS_Vn, Severity score at Visit n; G, group.

**Table 5:** Comparison of general health, physical functioning and physical role functioning at V3 and V4.

Dependent Variable	(I) (1 = G1, 2 = G2, 3 = G3)	(II) (1 = G1, 2 = G2, 3 = G3)	Mean Difference (I–II)	*p*-Value
V3_GH	2	1	23.25	0.0001
		3	18.91	0.0001
V3_PhF	2	1	8.167	0.003
		3	9.167	0.001
V4_GH	2	1	18.16	0.0001
		3	23.33	0.0001
V4_PhF	2	1	7.50	0.006
		3	6.50	0.022

A *p*-value < 0.05 was considered significant. Abbreviations: G, group; V, visit; GH, general health; PhF, physical functioning; PhRF, physical role functioning; SD, standard deviation.

**Table 6:** Comparison of changes rectal volume sensation for G1, G2, G3.

Comparison	95% Confidence Interval		
	Lower	Upper	*p*-Value
**G1**			
G1_V1_FS–G1_V2_FS	−14.612	−6.722	0.0001
G1_V1_FS–G1_V3_FS	−14.697	−6.970	0.0001
G1_V1_FS–G1_V4_FS	−16.091	−8.576	0.0001
G1_V1_T–G1_V2_T	−35.811	−16.189	0.0001
G1_V1_T–G1_V3_T	−24.952	−12.048	0.0001
G1_V1_T–G1_V4_T	−31.246	−16.088	0.0001
G1_V1_P–G1_V2_P	−36.687	−15.979	0.0001
G1_V2_P–G1_V3_P	−15.283	6.616	0.425
G1_V2_FS–G1_V3_FS	−3.650	3.317	0.923
G1_V2_T–G1_V3_T	1.303	13.697	0.019
G1_V3_T–G1_V4_T	−5.704	10.371	0.071
**G2**			
G2_V1_FS–G2_V3_FS	−20.961	−14.706	0.0001
G2_V3_FS–G2_V4_FS	−1.268	3.268	0.375
G2_V1_T–G2_V3_T	−26.432	−17.901	0.0001
G2_V3_T–G2_V4_T	−3.642	4.642	0.807
G2_V1_P–G2_V3_P	−43.260	−26.140	0.0001
G2_V3_P–G2_V4_P	−8.945	5.611	0.643
**G3**			
G3_V1_FS–G3_V2_FS	−13.642	−5.358	0.0001
G3_V1_FS–G3_V3_FS	−14.042	−6.291	0.0001
G3_V1_FS–G3_V4_FS	−15.994	−8.673	0.0001
G3_V1_T–G3_V2_T	−35.205	−14.129	0.0001
G3_V1_T–G3_V3_T	−31.255	−15.745	0.0001
G3_V1_T–G3_V4_T	−36.848	−19.486	0.0001
G3_V2_FS–G3_V3_FS	−4.159	2.826	0.699
G3_V2_T–G3_V3_T	−5.387	7.721	0.718
G3_V1_P vs. G3_V2_P	−38.501	−14.499	0.0001
G3_V2_P vs. G3_V3_P	−26.086	−2.914	0.016

A *p*-value < 0.05 was considered significant. Abbreviations: G—group, V—visit, FS—first sensation, T—tenesmus, P—pain, SD—standard deviation Intergroup results.

**Table 7:** Intergroup comparisons of changes in rectal volume sensation at V3.

Dependent Variable	(I) (1 = G1, 2 = G2, 3 = G3)	(II) (1 = G1, 2 = G2, 3 = G3)	Mean Difference (I–II)	*p* Value
V3_FS	1	2	−6.333	0.016
		3	2.000	1.000
	2	1	6.333	0.016
		3	8.333	0.001

A *p*-value < 0.05 was considered significant. Abbreviations: G, group; V, visit; GH, general health.
